# Upregulation of miR-196b-5p attenuates BCG uptake via targeting SOCS3 and activating STAT3 in macrophages from patients with long-term cigarette smoking-related active pulmonary tuberculosis

**DOI:** 10.1186/s12967-018-1654-9

**Published:** 2018-10-16

**Authors:** Yaoqin Yuan, Dongzi Lin, Long Feng, Mingyuan Huang, Huimin Yan, Yumei Li, Yinwen Chen, Bihua Lin, Yan Ma, Ziyu Ye, Yuezhi Mei, Xiaolin Yu, Keyuan Zhou, Qunzhou Zhang, Tao Chen, Jincheng Zeng

**Affiliations:** 1 Dongguan Key Laboratory of Medical Bioactive Molecular Developmental and Translational Research, Guangdong Provincial Key Laboratory of Medical Molecular Diagnostics, Guangdong Medical University, Dongguan, 523808 Guangdong China; 2 Dongguan Sixth People’s Hospital, Dongguan, 523008 Guangdong China; 3 Provincial Tuberculosis Reference Laboratory of Guangdong, Center for Tuberculosis Control of Guangdong Province, Guangzhou, 510630 China; 4 Department of Oral and Maxillofacial Surgery and Pharmacology, University of Pennsylvania School of Dental Medicine, Philadelphia, 19104 USA

**Keywords:** MiR-196b-5p, Cigarette smoking, Macrophages, STAT3 signaling pathway, Tuberculosis

## Abstract

**Background:**

Cigarette smoking (CS) triggers an intense and harmful inflammatory response in lungs mediated by alveolar and blood macrophages, monocytes, and neutrophils and is closely associated with prevalence of tuberculosis (TB). The risk of death in patients with long-term cigarette smoking-related pulmonary tuberculosis (LCS-PTB) is approximately 4.5 times higher than those with nonsmoking pulmonary tuberculosis (N-PTB). However, the mechanisms underlying the harmful inflammatory responses in the setting of LCS-PTB have not been well documented.

**Methods:**

28 cases LCS-PTB patients, 22 cases N-PTB patients and 20 cases healthy volunteers were enrolled in this study. Monocytes were isolated from peripheral blood mononuclear cells. Differentiated human MDM and U937 cell were prepared with M-CSF and PMA stimulation, respectively. The miR-196b-5p, *STAT1*, *STAT3*, *STAT4*, *STAT5A*, *STAT5B*, *STAT6*, *SOCS1* and *SOCS3* mRNA expression were detected by qRT-PCR. Western blot was performed according to SOCS1, SOCS3, and pSTAT3 expression. The mycobacterial uptake by MDMs from different groups of patients after *Bacillus Calmette*–*Guérin* (BCG) infection and agomir-196b-5p or antagomir-196b-5p transfection were used by flow cytometry analysis. Human IL-6, IL-10 and TNF-α levels on the plasma and cell culture supernatant samples were measured using ELISA. For dual-luciferase reporter assay, the SOCS3 3′-UTR segments, containing the binding elements of miR-196b-5p or its mutant versions were synthesized as sense and antisense linkers.

**Results:**

In this study, we found that IL-6, TNF-α production, SOCS3 mRNA expression were downregulated, while miR-196b-5p and STAT3 mRNA expression were upregulated in monocytes from LCS-PTB patients as compared to N-PTB patients. Meanwhile, we demonstrated that miR-196b-5p could target SOCS3 and activate STAT3 signaling pathway, which may possibly contribute to attenuation of BCG uptake and decrease in IL-6 and TNF-α production in macrophages.

**Conclusions:**

Our findings revealed that CS exposure regulates inflammatory responses in monocyte/macrophages from LCS-PTB patients via upregulating miR-196b-5p, and further understanding of the specific role of miR-196b-5p in inflammatory responses mightfacilitate elucidating the pathogenesis of LCS-PTB, thus leading to the development of new therapeutic strategies for PTB patients with long-term cigarette smoking.

**Electronic supplementary material:**

The online version of this article (10.1186/s12967-018-1654-9) contains supplementary material, which is available to authorized users.

## Background

Tuberculosis (TB) caused by infection of *Mycobacterium tuberculosis* (MTB) is one of the top 10 causes of death in developing countries [1]. The risk factors for TB include crowding, poor nutrition, alcoholism, race/ethnicity, socioeconomic status, diabetes mellitus, human immunodeficiency virus (HIV) infection and cigarette smoking [2, 3]. Cigarette smoking (CS) has been reported to be harmful to human lungs and associated with an increase in both mortality and morbidity of TB. It has been shown that the risk of death in patients with long-term cigarette smoking-related pulmonary tuberculosis (LCS-PTB) is approximately 4.5 times higher than those with nonsmoking pulmonary tuberculosis (N-PTB) [4]. Meanwhile, LCS-PTB patients always present more severe pulmonary lesions than N-PTB patients [5]. Additionally, previous studies have shown that CS triggers an intense inflammatory response in lungs mediated by increased infiltration of alveolar and blood macrophages, monoc**y**tes, and neutrophils that release large amounts of various inflammatory cytokines and chemokines [6–8]. However, the molecular mechanisms underlying such pulmonary inflammatory responses in the setting of LCS have not been well documented.

Host immune system plays a critical role in the containment and cure of TB infection. The major types of innate immune cells involved in TB infection include macrophages, dendritic cells (DCs), neutrophils and natural killer (NK) cells, whereas classically activated (M1) macrophages play a central role in eliminating MTB via multiple mechanisms, such as increasing the components of free reactive oxygen and nitrogen species, production of various pro-inflammatory cytokines, phagosome acidification, autophagy induction, and among others [9]. Although M1 macrophages are known as professional killers, MTB has adopted remarkable strategies to control phagosomal processing via inhibiting phagolysosome biogenesis and acidification processes [10]. Notably, MTB survival is greatly enhanced when macrophage polarity is shifted toward alternatively activated (M2) macrophage [11]. It has been reported that treatment of monocyte-derived macrophages (MDM) by cigarette smoke extract (CSE) results in the enrichment of IL-6, TNF-α, IL-1β and MMP-9, which are commonly associated with M1 macrophages, and simultaneously, the reduction of IL-4, IL-10, and IL-13, which are associated with M2 macrophages [12–15]. It has been shown that the signal transducer and activator of transcription 1 (STAT1) plays a major role in dictating M1 macrophage phenotype, whereas STAT3/STAT6 direct M2 macrophage polarization [16–18].

MicroRNAs (miRNAs), small noncoding RNAs of length 22 nucleotides, are important regulatory molecules and have been shown to be involved in the regulating development and functions of monocytes/macrophages [17]. Also, they have been found to be valuable novel biomarkers for cigarette smoking [19]. MiR-196b-5p has been identified in the most primitive hematopoietic stem cells (HSCs), the precursors of of monocytes/macrophage [20]. Zhang et al. has reported that the level of miR-196b in the serum of patients with active TB was 1285.93-fold higher than that in latent TB infection (LTBI), *Bacillus Calmette*–*Guérin* (BCG)-inoculated and un-inoculated individuals [21]. Our previous results demonstrated that miR-196b-5p promotes colorectal cancer stemness and chemo-resistance via activating STAT3 signaling [22]. Accumulating data have suggested that STAT3 is a major controller of the outcome of MTB infection [23]. However, the expression of miR-196b-5p in monocytes from LCS-PTB patients and its role in the modulation of macrophage-mediated inflammatory responses during MTB infection in humans is yet to be fully elucidated. In the present study, we found that miR-196b-5p was upregulated in monocytes from LCS-PTB patients and treatment of MDM with CSE significantly increased miR-196b-5p expression. Moreover, overexpressing miR-196b-5p attenuated the uptake of BCG by MDM via targeting SOCS3 and subsequent activation of STAT3 signaling. These findings suggest that miR-196b-5p might be a valuable novel biomarker and therapeutic target for LCS-PTB patients.

## Methods

### Subjects

28 cases long-term cigarette smoking PTB (LCS-PTB) patients, 22 cases nonsmoking PTB (N-PTB) patients and 20 cases healthy volunteers (HV) from Dongguan sixth People’s Hospital (Dongguan, China) were enrolled in this study based on clinical symptoms, chest X radiography, acid fast bacilli (AFB) staining of sputum smears, as we previously reported [24, 25]. Subjects with HIV infection, autoimmune diseases, diabetes, cancer, immunosuppressive treatment, or pulmonary tuberculosis history were excluded.

### Monocytes sorting

Peripheral blood mononuclear cells (PBMCs) were prepared to isolate monocytes using the MojoSort™ Human CD14 Nanobeads (BioLegend) according to the manufacturer’s instructions. The sorted monocytes were primary monocytes for RNA extraction.

### Cultures of human MDM and U937 cells

Human MDM and U937 (a monocytic cell line, obtained from Shanghai Chinese Academy of Sciences cell bank, China) cells were cultured in RPMI 1640 medium (Gibco) supplemented with 10% FBS (heat-inactivated, mycoplasma- and endotoxin-free), 10% M-CSF, 50 U/mL penicillin, 50 µg/mL streptomycin and 2 mM l-glutamine. Monocytes were isolated from PBMC as we previously described [24–26]. PBMC were seeded on 24-well plates at a density of 1.5 × 10^6^ cells/well and differentiated for 7 days, non-adherent cells were removed, and adherent cells were obtained as differentiated MDM (more than 95% of cells were CD14^+^, CD3^−^). Before experiments were performed, the cells were cultured at 5 × 10^5^ cells/well on 24-well plates, and 50 nM phorbol myristate acetate (PMA) was used to differentiate U937 cells for 24 h.

### RNA extraction and qRT- PCR

Total RNA was extracted using RNA Isolation Kit (Qiagen, USA), and reverse transcribed using the Revert Aid First Strand cDNA Synthesis Kit (Thermo, USA) according to the manufacturer’s protocol. This cDNA was amplified and quantified on CFX96 system (BIO-RAD, USA) using iQ SYBR Green (BIO-RAD, USA). The primers were provided in Additional file 1: Table S1. Primers for U6 and miR-196b-5p (Cat#: miRQ0001080) were synthesized and purified by RiboBio (Guangzhou, China) (http://www.sirna.cn/siteen/Products.aspx?id=181). QRT-PCR was performed according to a standard method, as we previously described [22]. U6 or GAPDH was used as endogenous controls. Relative expressions were calculated with the comparative threshold cycle (2^−ΔΔCt^) method.

### Flow cytometric analysis

The isolated PBMCs were resuspended in 2% FBS-PBS, and then stained with indicated antibodies against human CD3 (HIT3α, BioLegend), CD14 (63D3, BioLegend), IL-6 (UV4, BioLegend), IL-10 (JES5-16E3, eBioscience), TNF-α (Mab11, BioLegend), and detected by flow cytometry (BD FACS Calibur II, San Jose, CA, USA) and analyzed using FlowJo 7.6 software (TreeStar Inc., USA) as we previously described [24–26]. Mouse IgG1 (MOPC-21), IgG2b (27–35), IgG2a (G155–178), and rat IgG2b (κ) were used as isotype control.

### Exposure of cells to BCG

For testing the effects of miR-196b-5p on mycobacterial uptake in vitro, MDM or U937 macrophages were infected with green fluorescent protein (GFP) tagged BCG at the multiplicity of infection (MOI) of 10 for 6 h, then washed and cultivated for 5 days. Bacterial uptake was evaluated by measuring the percentage and median fluorescence intensity (MFI) of GFP^+^ MDM (BCG uptake macrophages) by flow cytometric analysis.

### MiRNAs, siRNA, lentiviral particles and transfection

The agomir-196b-5p (Cat#: miR40001080), antagomir-196b-5p (Cat#: miR30001080), small interfering RNA (siRNA) for SOCS3 (SOCS3-RNAi) and respective control RNA were synthesized and purified by RiboBio, as we previously described [22]. SOCS-3 lentiviral activation particles (SOCS3-LAC, sc-400455-LAC) was purchased from Santa Cruz. Transfection of miRNAs, siRNA were performed using Lipofectamine 3000 (Life Technologies, USA) according to the manufacturer’s instructions.

### Western blotting analysis

Nuclear/cytoplasmic fractionation was separated using Cell Fractionation Kit (Cell Signaling Technology, USA) and the whole cell lysates were extracted using RIPA Buffer (Cell Signaling Technology) according to the manufacturer’s instructions. Western blot was performed according to a standard method, as we previously described [22]. Proteins were visualized using ECL reagents (Pierce, USA). Antibodies against SOCS1, SOCS3, STAT3 and pSTAT3 were purchased from Cell Signaling Technology.

### Dual-luciferase reporter assay

For dual-luciferase reporter assay, the SOCS3 3′-UTR segments, containing the binding elements of miR-196b-5p or its mutant versions were synthesized as sense and antisense linkers. The wild-type and mutated 3′-UTR fragments were then cloned into the downstream of luciferase reporter gene of pmirGLO vectors (Promega, Madison, WI, USA). PmirGLO-Report-WT-SOCS1 (harboring wild-type 3′-UTR) and pmirGLO-Report-Mut-SOCS1 (harboring mutant 3′-UTR) were generated. The specificity of miR-196b-5p targeting SOCS3 mRNA was ascertained by co-transfection of miR-196b-5p mimic or inhibitor (RiboBio, Guangzhou, China) and pmirGLO-Report-SOCS1/Mut into U937 cells. Additionally, as we previously described [22], relative luciferase activity of STAT3 was detected using pSTAT3 reporter luciferase plasmid (Promega). Luciferase and Renville signals were measured 36 h after transfection using a Dual Luciferase Reporter Assay Kit (Promega, Madison, WI, USA) according to the manufacturer’s protocol as we previously described [22].

### Enzyme-linked immunosorbent assay (ELISA)

Cytokines were measured in the plasma and cell culture supernatant samples using human IL-6, IL-10 and TNF-α ELISA Kit (Ray Biotech, Atlanta, USA) according to the manufacturer’s instructions. Cell culture supernatant was diluted 1:10. Plasma was used as an undiluted specimen.

### Statistical analysis

All analyses were performed using GraphPad Prism version 5.0 software (GraphPad Software Inc., San Diego, CA, USA). Data are presented as mean ± SEM (standard error of the mean, SEM). Student’s t-test or ANOVA (analysis of variance, ANOVA) was employed to compare the differences of measured data. A *P* value of less than 0.05 (95% confidence interval) was considered with statistical significance.

## Results

### Characteristics of the subjects included in the study

Among all prospectively enrolled subjects, 28 cases were LCS-PTB patients, 22 cases were N-PTB patients and 20 cases were healthy volunteers (HV). The demographic and clinical characteristics for all study subjects were shown in Table 1. No significant differences regarding age and gender were noted among LCS-PTB patients, N-PTB patients, and HV volunteers. Among LCS-PTB patients, 100% (28/28) were positive for acid-fast bacilli (AFB) smear-MTB and tuberculin skin test (TST) (induration diameter ≥ 10 mm), and long-term cigarette smoking (> 7 years), while 53.6% (15/28) of them were newly diagnosed LCS-PTB patients. Among N-PTB patients, all of them were AFB smear- MTB and TST (induration diameter ≥ 10 mm) positive, while 54.5% (12/22) of N-PTB patients were newly diagnosed patients. Both LCS-PTB patients and N-PTB patients had taken an ATD treatment.

**Table 1:** Demographics of subjects included in the study

Group	LCS-PTB (n = 28)	N-PTB (n = 22)	HV (n = 20)
Age, years	43.33 ± 2.19	41.17 ± 2.71	39.92 ± 2.68
Male/female	18/10	14/8	12/8
AFB smear-positive, n (%)	28 (100)	22 (100)	0 (0)
Smoking (ever), ≥ 7 years, n (%)	28 (100)	0 (0)	0 (0)
New/Relapsed, n/n	15/13	12/10	–
Fever, n (%)	0 (0)	0 (0)	0 (0)
TST, ≥ 10 mm, n (%)	28 (100)	22 (100)	0 (0)
ATD treatment	28 (100)	22 (100)	0 (0)

*TST* tuberculin skin test, *AFB* acid-fast bacilli, *ATD* anti-tuberculosis drug

### Blood monocytes were elevated in LCS-PTB patients

**Fig. 1:**
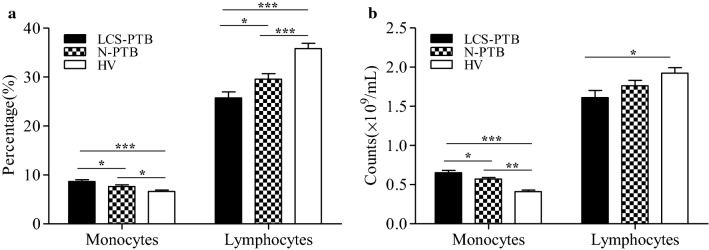
Monocytes were elevated in LCS-PTB patients. The percentage (**a**) and count (**b**) of blood monocytes and lymphocytes among LCS-PTB patients, N-PTB patients and HV groups were detected by laboratory tests. Data were presented as mean ± SEM. *P < 0.05; **P < 0.01; ***P < 0.001

Monocytes originated from the bone marrow represent approximately 10% of human leucocytes in the bloodstream and have traditionally been regarded as the progenitors of tissue macrophages. Monocytes could differentiate into macrophages and mediate a wide range of immunologic response processes, including reactions against TB infection [27–29]. Veenstra et al. and our group have previously shown that the counts and percentage of monocytes in the peripheral blood were significantly higher in active PTB patients than that in HV volunteers [25, 30]. Herein, our laboratory tests also revealed an obvious rise in the percentage and counts of monocytes in peripheral blood of LCS-PTB and N-PTB patients as compared to that in HV volunteers (Fig. 1a, b). Additionally, both percentage and counts of monocytes were even higher in peripheral blood of LCS-PTB patients than of N-PTB patients (Fig. 1a, b). However, our results showed that that the total count of lymphocytes was significantly decreased in peripheral blood of LCS-PTB patients as compared with those of both N-PTB patients and HV volunteers (Fig. 1a). These results suggest that LCS-PTB and N-PTB patients may have different systemic immune responses to TB infection.

### IL-6 and TNF-α were suppressed in LCS-PTB patients

**Fig. 2:**
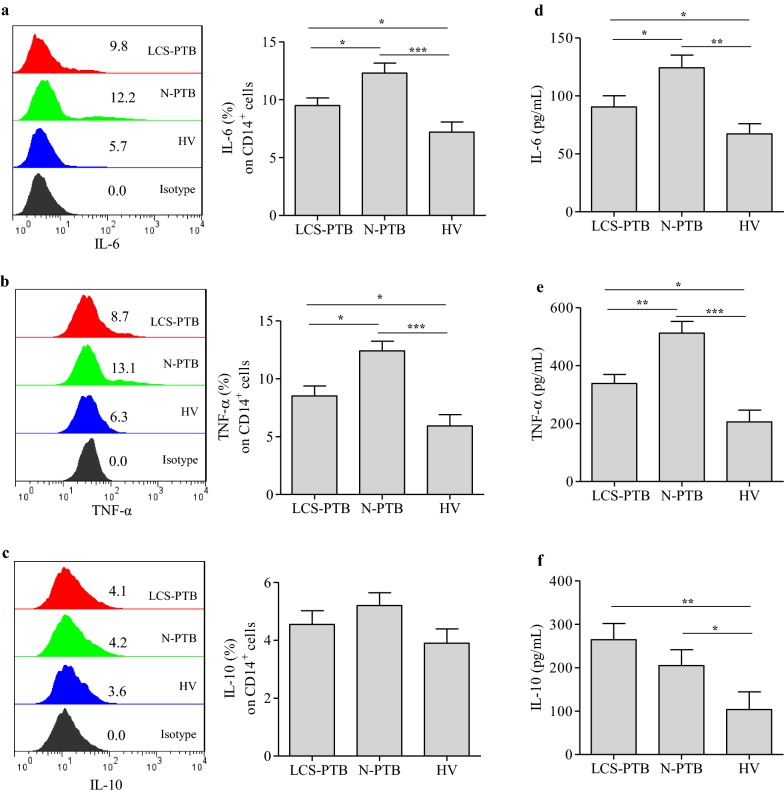
IL-6 and TNF-α were suppressed on monocytes from LCS-PTB patients. The production of IL-6 (**a**), TNF-α (**b**) and IL-10 (**c**) of monocytes among LCS-PTB patients, N-PTB patients, and HV groups were detected by FCM analysis. And the serum IL-6 (**d**), TNF-α (**e**) and IL-10 (**f**) levels among LCS-PTB patients, N-PTB patients, and HV groups were detected by ELISA. Data were presented as mean ± SEM. *P < 0.05; **P < 0.01; ***P < 0.001

To further explore the innate immune responses in LCS-PTB patients, the IL-6, TNF-α, and IL-10 production in peripheral monocytes were detected by flow cytometric analysis. Our results showed that the percentage of CD14^+^ monocytes positive for both IL-6 and TNF-αwas significantly increased in peripheral blood of LCS-PTB and N-PTB patients as compared to that of HV volunteers, respectively (Fig. 2a, b). Interestingly, in comparison to N-PTB patients, LCS-PTB patients with an elevated number of monocytes in peripheral blood had a relatively lower percentage of IL-6- or TNF-α-positive CD14^+^ monocytes (Figs. 1a, b, 2a, b). However, no obvious differences in the percentage of IL-10-positiveCD14^+^ monocytes in the peripheral blood were noticed among LCS-PTB patients, N-PTB patients, and HV volunteers (Fig. 2c). In parallel, we examined the serum levels of IL-6, TNF-α, and IL-10 by ELISA. Our results showed that the serum levels of IL-6, TNF-α, and IL-10 were significantly increased in both LCS-PTB and N-PTB patients in comparison to those of HV volunteers (Fig. 2d–f), which was in consistent with previous findings by us and others [25, 31, 32]. Similarly, we found that the serum levels of IL-6 and TNF-α of LCS-PTB patients were significantly lower than those of N-PTB patients (Fig. 2d, e). These results suggest that LCS-PTB patients exhibited suppressive systemic immune responses mediated by peripheral monocytes as compared with N-PTB patients.

### miR-196b-5p was upregulated in peripheral monocyte from LCS-PTB patients

**Fig. 3:**
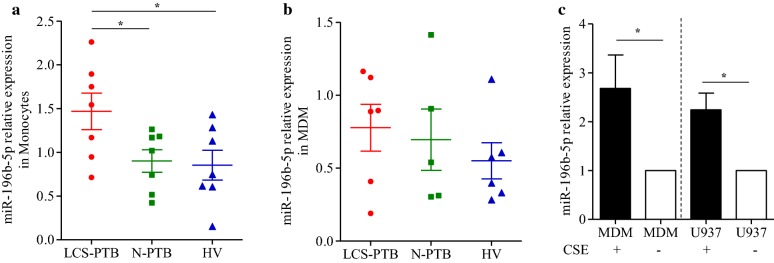
miR-196b-5p was upregulated on monocyte from LSC-PTB patients. The relative expression of miR-196b-5p on sorted CD14^+^ monocyte (**a**) and MDM (**b**) among LCS-PTB patients, N-PTB patients and HV groups were detected by qRT-PCR. Furthermore, miR-196b-5p expression on MDM or differentiated U937 cells were detected by qRT-PCR after cigarette smoke extract (CSE) treatment (**c**). Data were presented as mean ± SEM. *P < 0.05; **P < 0.01; ***P < 0.001

Our previous results demonstrated that miR-196b-5p could activate STAT3 signaling pathway via targeting negative regulators SOCS1 and SOCS3 [22], which plays a key role in the differentiation of monocytes into macrophages [16, 23]. We then determined whether there are any changes in the expression of miR-196b-5p in primary peripheral monocytes and MDMs. Our results showed that the relative expression of miR-196b-5p was significantly upregulated in monocytes from LCS-PTB patients as compared to that of N-PTB patients or HV volunteers (Fig. 3a). However, no significant differences in miR-196b-5p expression were noticed in MDMs among LCS-PTB patients, N-PTB patients or HV groups (Fig. 3b). However, the expression of miR-196b-5p was upregulated in MDMs or differentiated U937 cells in response to CSE treatment (Fig. 3c).

### *STAT3* mRNA expression was upregulated while *SOCS3* mRNA expression was downregulated in peripheral monocytes from LCS-PTB patients

**Fig. 4:**
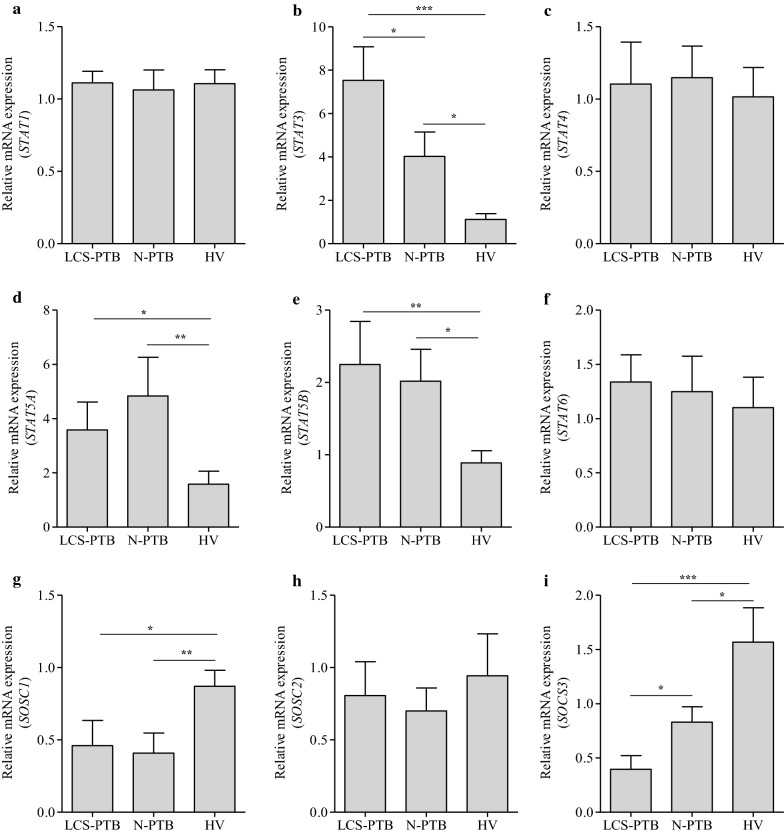
*STAT3* mRNA expression was upregulated, while *SOCS3* mRNA expression was downregulated on monocytes from LSC-PTB patients. The relative mRNA expression of *STAT1* (**a**), *STAT3* (**b**), *STAT4* (**c**), *STAT5A* (**d**), *STAT5B* (**e**), *STAT6* (**f**)*, SOCS1* (**g**), *SOCS2* (**h**) and *SOCS3* (**i**) on sorted CD14^+^ monocyte among LCS-PTB patients, N-PTB patients and HV groups were detected by qRT-PCR. Data were presented as mean ± SEM. *P < 0.05; **P < 0.01; ***P < 0.001

STAT pathways were among the most important signaling pathways in regulating the function of monocytes or macrophages. To dissect immune responses mediated by STAT signaling pathways in peripheral monocytes from LCS-PTB patients, N-PTB patients, and HV volunteers, the mRNA expression of *STAT1*, *STAT3*, *STAT4*, *STAT5A*, *STAT5B* and *STAT6* was detected by qRT-PCR (Fig. 4a–f). Our results indicated that *STAT3*, *STAT5A*, *STAT5B* mRNAs were upregulated in monocytes from both LCS-PTB and N-PTB patients as compared to those of HV volunteers (Fig. 4b, d, e). Interestingly, only *STAT3* mRNA expression was more pronounced in monocyted from LCS-PTB patients than that of N-PTB patients (Fig. 4b), whereas no significant differences in the mRNA expression of *STAT1*, *STAT4*, *STAT5A*, *STAT5B* and *STAT6* were noticed in monocytes from LCS-PTB and N-PTB patients (Fig. 4a–f).

SOCSs play a key role in the regulation of STAT3 signaling pathways. Previous studies showed that SOCS1, SOCS2, and SOCS3 were upregulated in patients with active PTB [33–35] and SOCS3 attenuated anti-inflammatory effects of IL-6 in macrophages [36]. We then detected *SOCS1*, *SOCS2* and SOCS3 mRNA expressions in peripheral monocytes from LCS-PTB and N-PTB patients. Our results indicated that *SOCS1* and *SOCS3* mRNA expression were downregulated in monocytes from both LCS-PTB and N-PTB patients as compared to that of HV volunteers (Fig. 4g, i). However, an even lower expression level of *SOCS3* mRNA was observed in monocytes from LCS-PTB patients than that in those of N-PTB patients (Fig. 4i). These data suggest that downregulation of SOCS3 and activation STAT3 may play a potential role in suppressing the pro-inflammatory responses in peripheral monocytes of LCS-PTB patients.

### miR-196b-5p inhibited BCG uptake by macrophages

**Fig. 5:**
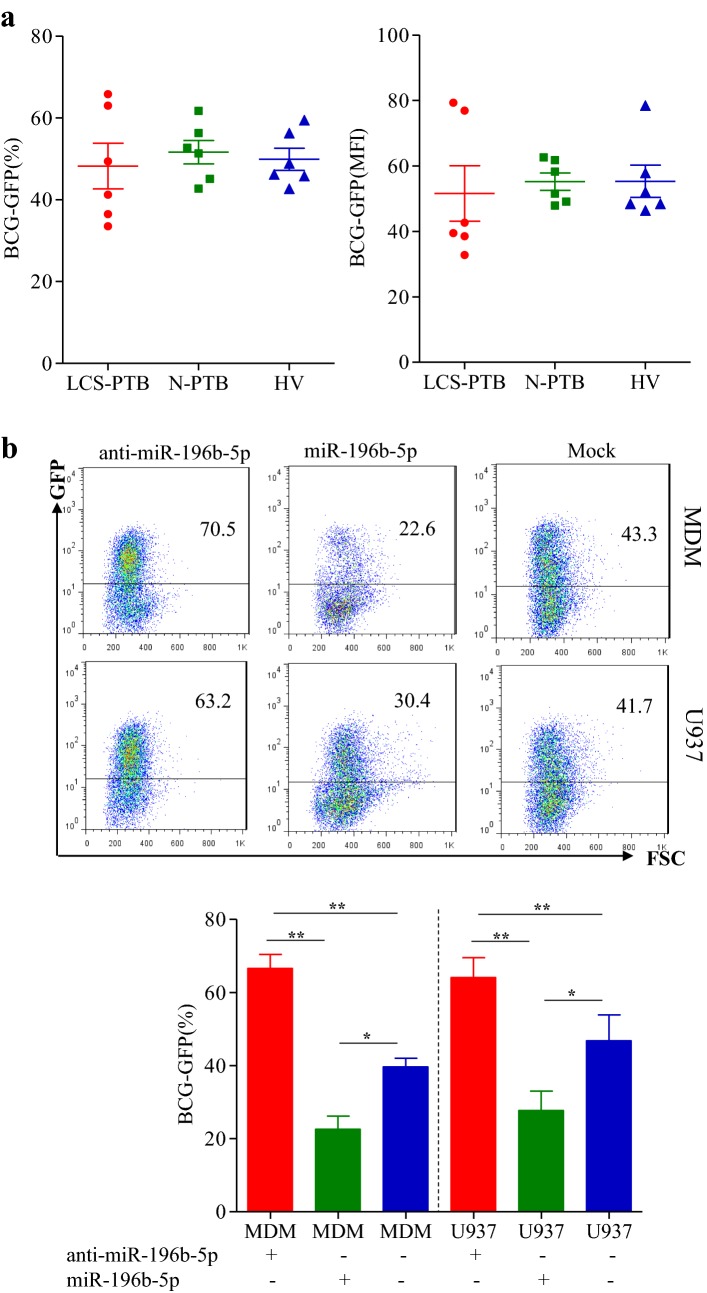
miR-196b-5p inhibited BCG uptake by macrophages. GFP tagged BCG was used to detect mycobacterial uptake by macrophages. The percentage and median fluorescence intensity (MFI) of GFP^+^ MDM (BCG uptake macrophages) among LCS-PTB patients, N-PTB patients and HV groups were detected by FCM analysis (**a**). And the BCG uptake by MDMs or differentiated U937 cells was detected after exogenously miR-196b-5p and anti-miR-196b-5p transfected (**b**). Data were presented as mean ± SEM. *P < 0.05; **P < 0.01; ***P < 0.001

In order to further explore the functional role of miR-196-5b in monocytes of LCS-PTB patients, MDMs from 6 cases of LCS-PTB patients, N-PTB patients, and HV control volunteers, respectively were obtained. First, we compared mycobacterial uptake by MDMs from different groups of patients after BCG infection using flow cytometry analysis. After 5 days of BCG infection, the BCG uptake ability by MDMs had no significant differences among LCS-PTB patients, N-PTB patients, and HV volunteers (Fig. 5a). However, upregulation of miR-196b-5p with agomir-196b-5p inhibited BCG uptake, while downregulation of miR-196b-5p with antagomir-196b-5p (anti-miR-196b-5p) promoted BCG uptake in MDMs or differentiated U937 cells (Fig. 5b). These results suggested that overexpression of miR-196b-5p can attenuate the capability of macrophages to phygocytose BCG.

### miR-196b-5p inhibited IL-6, TNF-α production in macrophages via activating STAT3 signaling pathway

**Fig. 6:**
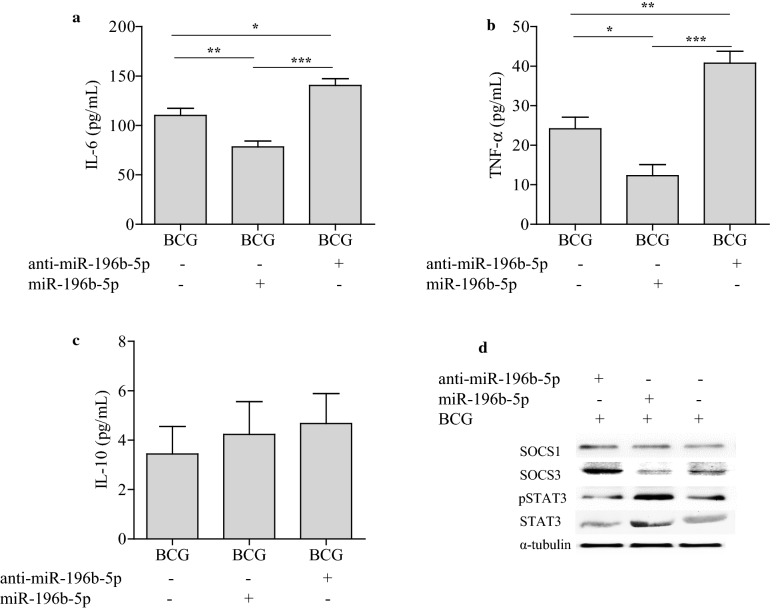
miR-196b-5p inhibited IL-6, TNF-α production on macrophages via activating STAT3 signaling pathway. The supernatant IL-6 (**a**), TNF-α (**b**) and IL-10 (**c**) levels on differentiated U937 cells after exogenously miR-196b-5p, anti-miR-196b-5p transfected were detected by ELISA. And SOCS1, SOCS3, STAT3 and pSTAT3 expression on differentiated U937 cells after exogenously miR-196b-5p, anti-miR-196b-5p transfected were detected by western blotting analysis (**d**). Data were presented as mean ± SEM. *P < 0.05; **P < 0.01; ***P < 0.001

Beside impairment of macrophage phagocytosis of BCG, we also found that upregulation of miR-196b-5p decreased while downregulation of miR-196b-5p increased the production of pro-inflammatory cytokines IL-6 and TNF-α, but not the anti-inflammatory cytokine IL-10, in differentiated U937 cells after BCG infection (Fig. 6a–c). In consistent with above findings, pSTAT3 was upregulated while SOCS3 was downregulated following upregulation of miR-196b-5p in macrophages (Fig. 6d). Those results suggest that upregulation of miR-196b-5p led to reduced secretion of pro-inflammatory cytokines in macrophages possibly by downregulating *SOCS3* and activating STAT3 signaling pathway.

### miR-196b-5p promoted STAT3 signaling pathway via targeting SOCS3

**Fig. 7:**
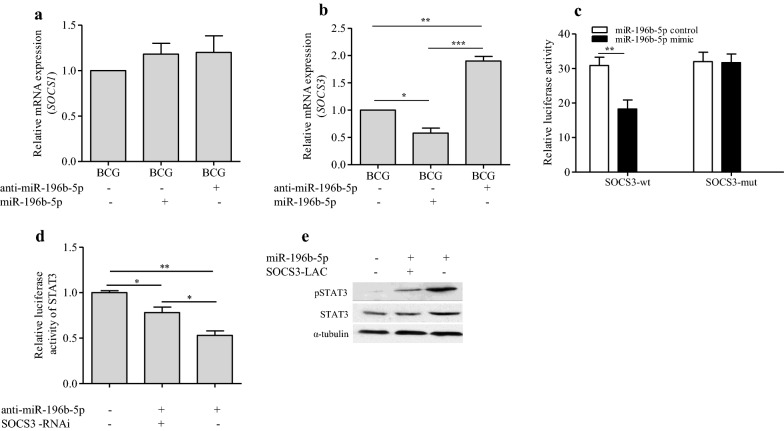
miR-196b-5p promoted STAT3 signaling pathway via targeting SOCS3. The relative mRNA expression of *SOCS1* (**a**) and *SOCS3* (**b**) on differentiated U937 cells after exogenously miR-196b-5p, anti-miR-196b-5p transfected were detected by qRT-PCR. Luciferase assay also showed that upregulation of miR-196b-5p attenuated the reporter activity driven by the wildtype 3′-UTRs, but not by the mutant 3′-UTRs of *SOCS3* transcripts within miR-196b-5p-binding seed regions in U937 cells (**c**). Silencing of SOCS3 with SOCS3-RNAi rescued the STAT3 activity repression in miR-196b-5p-silencing U937 cells (**d**). pSTAT3 were downregulated after overexpressing of SOCS3 in miR-196b-5p-overexpressing U937 cells with SOCS3-LAC (**e**). Data were presented as mean ± SEM. *P < 0.05; **P < 0.01; ***P < 0.001

Our previous studies showed that miR-196b-5p targets 3′-UTRs of *SOCS1* and *SOCS3* mRNA to promote STAT3 signaling pathway in colorectal cancer cells [22]. We then determined whether miR-196b-5p could promote STAT3 signaling pathway in macrophages through SOCS1 or SOCS3. *SOCS1* and *SOCS3* mRNA expressions were detected by qRT-PCR following upregulation or downregulation of miR-196b-5p in differentiated U937 cells. Our data showed that upregulation of miR-196b-5p inhibited while downregulation of miR-196b-5p increased *SOCS3* but not *SOCS1* mRNA expression in macrophages, suggesting that SOCS3 may also be a target of miR-196b-5p in macrophages (Fig. 7a, b). In addition, luciferase assay also showed that upregulation of miR-196b-5p attenuated the reporter activity driven by the wildtype 3′-UTRs of *SOCS3* transcripts, but not by mutant 3′-UTRs of *SOCS3* transcripts, within miR-196b-5p-binding seed regions in U937 cells (Fig. 7c). Notably, we also found that silencing of SOCS3 with SOCS3-RNAi rescued the STAT3 activity repression in miR-196b-5p-silencing U937 cells (Fig. 7d). Additionally, total STAT3, especially phosphorylated STAT3 were downregulated after overexpressing of SOCS3 with SOCS3-LAC in miR-196b-5p-overexpressing U937 cells (Fig. 7e). Collectively, these results suggest that miR-196b-5p activates STAT3 signaling pathway possibly via targeting SOCS3.

## Discussion

Inflammatory responses are an essential part of innate immune responses to pathogens. Monocytes or macrophages were important effectors and regulators in innate immunity. Numerous studies, including our previous reports, showed an increased number of circulating blood monocytes in TB patients [25, 30, 37, 38]. However, blood monocyte-derived macrophages in PTB patients exhibit impaired phagocytic capacity [39]. MTB infection recruited the inflammatory monocytes or macrophages into lungs, which contribute to granuloma formation [40, 41] whereby IL-6, and TNF-α play an important role [25, 42–44]. Herein, we also found that IL-6- and TNF-α-positive CD14^+^ monocytes and their serum levels were significantly increased in peripheral blood of both LCS-PTB and N-PTB patients as compared with those in HV volunteers, which is in consistent with the previous results found in adult but not childhood TB patients [25, 43, 45–48]. However, the percentage of IL-6- and TNF-α-positive CD14^+^ monocytes were relatively lower in peripheral blood of LCS-PTB patients (cigarette smoking > 7 years) than that of N-PTB patients, although the absolute count and percentage of monocytes increased in both LCS-PTB and N-PTB patients. In parallel, we found an elevated level of IL-6 and TNF-α in serum of both LCS-PTB and N-PTB patients as compared to that of HV volunteers, while the elevation of IL-6 and TNF-α in serum of LCS-PTB was less pronounced than that of N-PTB patients. Functionally, we showed that MDMs of LCS-PTB patients exhibited a lower uptake of BCG or impaired phagocytosis of BCG. These results support the notion that long-term cigarette smoking may lead to impaired phagocytosis of BCG by macrophages due to attenuated pro-inflammatory responses.

Aberrant activation of STAT3 signaling pathway was linked to immune disorders and inflammatory response of monocytes or macrophages. Human STAT family currently consisted of seven members, STAT1, STAT2, STAT3, STAT4, STAT5A, STAT5B, STAT6. In this study, we showed that *STAT3*, *STAT5A*, *STAT5B* mRNAs were upregulated in peripheral CD14^+^ monocytes from LCS- and N-PTB patients as compared with those of HV controls. Moreover, we have demonstrated, for the first time to our knowledge, that the increase in *STAT3* mRNA expression and pSTAT3 expression were less pronounced in CD14^+^ monocytes and MDM from LCS-PTB patients than in those from N-PTB patients, which were inversely correlated with the expression levels of *SOCS3* mRNA and protein. Emerging evidence indicates that deregulation of negative regulators of STAT3 signaling pathway including tyrosine phosphatase and SOCS families plays a crucial role in the activation of STAT3 signaling [49–51]. Therefore, our results suggest that SOCS3 is involved in the regulation of STAT3 activation in peripheral CD14^+^ monocytes or MDMs from LCS-PTB patients.

Most recently, the biological role and clinical significance of miR-196b-5p have been extensively studied. Overexpression of miR-196-5p has been reported in the hematologic malignancies, solid tumors, coronary artery disease and Ebola virus infection [22, 52–55]. Zhang et al. has reported that the serum level of miR-196b in active TB patients was higher than that in latent TB infection (LTBI), BCG-inoculated and un-inoculated individuals [21]. In the present study, we demonstrated that miR-196b-5p was upregulated in peripheral monocytes from LCS-PTB patients as compared with that in those from N-PTB patients. Additionally, we showed CSE and PM2.5 (unpublished data) increased miR-196b-5p expression in MDMs and differentiated U937 cells. Overexpression of miR-196b-5p downregulated the expression of SOCS3 mRNA while simultaneously activated STAT3 signaling pathway in macrophages.

## Conclusions

In summary, our findings support the notion that long-term cigarette smoking may lead to upregulated expression of miR-196b-5p, which then contributes to targeted inhibition of SOCS3 and activation of STAT3 signaling pathway, and subsequently, the impaired phagocytosis or elimination of BCG by macrophages due to their attenuated pro-inflammatory responses. Therefore, miR-196b-5p may represent a novel biomarker and therapeutic target for the treatment of LCS-PTB patients.

## Additional file

**Additional file 1: Table S1.** A list of primers used in the investigated genes.

## Abbreviations

CS: cigarette smoking
TB: tuberculosis
LCS-PTB: long-term cigarette smoking-related pulmonary tuberculosis
N-PTB: nonsmoking pulmonary tuberculosis
HV: healthy volunteers
PBMC: peripheral blood mononuclear cells
BCG: Bacillus Calmette–Guérin
MTB: mycobacterium tuberculosis
MDM: monocyte-derived macrophages
CSE: cigarette smoke extract
ELISA: enzyme-linked immunosorbent assay
