# Supplementary material for: Upregulation of miR-196b-5p attenuates BCG uptake via targeting SOCS3 and activating STAT3 in macrophages from patients with long-term cigarette smoking-related active pulmonary tuberculosis
Source: J Transl Med. 2018 Oct 16;16:284. doi: 10.1186/s12967-018-1654-9 (PMC6192289; doi:10.1186/s12967-018-1654-9)
Supplement: Supplementary file 1 — Additional file 1: Table S1. A list of primers used in the investigated genes. [file 12967_2018_1654_MOESM1_ESM.doc]

**Additional file 1: Table S1. A list of primers used in the investigated genes**

| **Gene** | **Primers** |
| --- | --- |
| *STAT1* | F 5’- CATTCACATGGGTGGAGCG-3’ |
|  | R 5’- GGGTTCAACCGCATGGAAG-3’ |
| *STAT3* | F 5’- TGCTGGTGACTGGATAGCAG-3’ |
|  | R 5’- CTCCTTGGAAGGTGCTGAAG-3’ |
| *STAT4* | F 5’- GGAAATTCGGCATCTGTTGGCC-3’ |
|  | R 5’- TTCTCTTTGGAAACACGACCTAACTGT-3’ |
| *STAT5A* | F 5’- GAAGCTGAACGTGCACATGAATC-3’ |
|  | R 5’- GTAGGGACAGAGTCTTCACCTGG-3’ |
| *STAT5B* | F 5’- AGTTTGATTCTCAGGAAAGAATGT-3’ |
|  | R 5’- TCCATCAACAGCTTTAGCAGT-3’ |
| *STAT6* | F 5’- GGCCACTTTCAGACAAATACTTCAAGGA-3’ |
|  | R 5’- TGCAGCCTCCGCAAGCCT-3’ |
| *SOCS1* | F 5’-CACATGGTTCCAGGCAAGTA-3’ |
|  | R 5’-CTACCTGAGCTCCTTCCCCT-3’ |
| *SOCS2* | F 5’-GGAGGACGGATGACAAAGTC-3’ |
|  | R 5’-AGACACTCTCCGGACTGAGG-3’ |
| *SOCS3* | F 5’-CAAGGACGGAGACTTCGATT-3’ |
|  | R 5’-AACTTGCTGTGGGTGACCAT-3’ |
| *GAPDH* | F 5’-ATTCCACCCATGGCAAATTC-3’ |
|  | R 5’-TGGGATTTCCATTGATGACAAG-3’ |

F = Primer forward; R = Primer reverse.
